# Prone versus Supine Position Ventilation in Adult Patients with Acute Respiratory Distress Syndrome: A Meta-Analysis of Randomized Controlled Trials

**DOI:** 10.1155/2020/4973878

**Published:** 2020-11-30

**Authors:** Zanfeng Cao, Zhanzheng Yang, Zijing Liang, Qingyan Cen, Zuopeng Zhang, Hengrui Liang, Rong Liu, Liangbo Zeng, Yubao Xie, Youping Wang

**Affiliations:** ^1^Department of Emergency Room, The First Affiliated Hospital of Guangzhou Medical University, Guangzhou 510120, China; ^2^Guangzhou Medical University, Guangzhou 510000, China; ^3^Department of Thoracic Surgery, The First Affiliated Hospital of Guangzhou Medical University, Guangzhou Institute of Respiratory Health (GIRH), State Key Laboratory of Respiratory Disease, National Clinical Research Center for Respiratory Diseases, Guangzhou 510120, China

## Abstract

The purpose of this meta-analysis was to compare the efficacy and safety of prone versus supine position ventilation for adult acute respiratory distress syndrome (ARDS) patients. The electronic databases of PubMed, Embase, and the Cochrane Library were systematically searched from their inception up to September 2020. The relative risks (RRs) and weighted mean differences (WMDs) with corresponding 95% confidence intervals (CIs) were employed to calculate pooled outcomes using the random-effects models. Twelve randomized controlled trials that had recruited a total of 2264 adults with ARDS were selected for the final meta-analysis. The risk of mortality in patients who received prone position ventilation was 13% lower than for those who received supine ventilation, but this effect was not statistically significant (RR: 0.87; 95% CI: 0.75–1.00; *P* = 0.055). There were no significant differences between prone and supine position ventilation on the duration of mechanical ventilation (WMD: −0.22; *P* = 0.883) or ICU stays (WMD: –0.39; *P* = 0.738). The pooled RRs indicate that patients who received prone position ventilation had increased incidence of pressure scores (RR: 1.23; *P* = 0.003), displacement of a thoracotomy tube (RR: 3.14; *P* = 0.047), and endotracheal tube obstruction (RR: 2.45; *P* = 0.001). The results indicated that prone positioning during ventilation might have a beneficial effect on mortality, though incidence of several adverse events was significantly increased for these patients.

## 1. Introduction

Acute respiratory distress syndrome (ARDS) is a serious disorder in critically ill patients that is characterized by disrupted endothelial barriers, abnormal alveolar epithelium, pulmonary vascular permeability, and protein-rich pulmonary edema [1]. The mortality of ARDS remains high, and the pooled mortality rate in our meta-analysis was 43%, ranging from 26% to 58% [2–4]. The mortality of severe ARDS exceeds 60%, although low-volume, low-pressure ventilation strategies have been employed to reduce ventilator-induced lung injury [5–8]. Therefore, efforts to limit mechanical lung injury during invasive ventilation are widely used for improving survival in ARDS patients [7].

Prone position ventilation has been adopted in ARDS patients in order to improve oxygenation and lung recruitment [9]. The mechanisms included improved ventilation-perfusion matching, end-expiratory lung volume, and ventilator-induced lung injury [10, 11]. Numerous randomized controlled trials (RCTs) comparing prone position ventilation with supine positioning have been conducted, and the results are varied. Uncertainty remains regarding the differences in efficacy and safety for prone versus supine positioning in ventilation of adults with ARDS. Therefore, this meta-analysis, based on published RCTs, was carried out to evaluate the efficacy and safety of prone versus supine position ventilation in patients with ARDS.

## 2. Materials and Methods

### 2.1. Data Sources, Search Strategy, and Selection Criteria

This study was performed in concordance with the Preferred Reporting Items for Systematic Reviews and Meta-Analysis (PRISMA) Statement [12]. RCTs investigating the efficacy and safety of prone versus supine position ventilation in patients with ARDS were eligible for this meta-analysis. PubMed, Embase, and the Cochrane Library were searched from their inception up to September 2020, and the following searching terms were combined by AND or OR: body posture, body position, prone position, prone positioning, ARDS, respiratory failure, and lung injury. The reference lists of the retrieved studies were also reviewed manually to identify any new or additional studies.

Two authors independently conducted the study selection, and any conflicts were settled by discussion until a consensus was reached. The inclusion criteria included: (1) patients, adults with ARDS; (2) intervention, prone position; (3) control, supine position; (4) outcomes, efficacy outcomes including mortality, mechanical ventilation duration, and ICU stays, and the safety outcomes, including any adverse events reported ≥2 studies; and (5) study design: RCT.

### 2.2. Data Collection and Quality Assessment

Data abstraction and quality assessment were carried out by two authors, and any disagreements were settled by an additional author. The collected variables included: first author's surname, publication year, country, sample size, mean age, percentage of male patients, mean partial pressure of arterial oxygen (PaO_2_), fractional concentration of inspired oxygen (FIO_2_), mean positive end-expiratory pressure (PEEP), mean FIO_2_, duration of ARDS, duration of prone positioning, protective lung ventilation, and reported outcomes. The Jadad scale, taking into consideration randomization, blinding, allocation concealment, withdrawals and dropouts, and use of intention-to-treat analysis, was applied to assess the quality of included studies [13]. The Jadad scale scores ranged from 0 to 7; studies with a score ≥5 were defined as high quality.

### 2.3. Statistical Analysis

The treatment effectiveness of prone versus supine position ventilation were assigned as dichotomous and continuous data, and the relative risks (RRs) and weighted mean differences (WMDs) with 95% confidence intervals (CIs) were calculated before data pooling. The pooled effect estimates were calculated and applied to the random-effects model (the DerSimonian–Laird method) [14, 15]. Heterogeneity tests were conducted using *I*^*2*^ and *Q* statistic, and *I*^*2*^ ≥ 50.0% or *P* < 0.10 was regarded as significant heterogeneity [16, 17]. Sensitivity analyses for mortality, mechanical ventilation duration, and ICU stays were conducted to assess the robustness of pooled results [18]. The subgroup analyses for mortality were then performed according to sample size, mean age, percentage male, duration of intervention, protective lung ventilation, and study quality. The differences between subgroups were assessed by using the interaction *P* test [19]. A funnel plot, Egger's test, and Begg's test were used to assess publication bias for mortality [20, 21]. All the pooled effects were determined using the Z-test, and two-sided *P* < 0.05 was considered statistically significant. STATA software was used for all of statistical analyses in this study (version 12.0, Stata Corporation, College Station, TX, USA).

## 3. Results and Discussion

### 3.1. Literature Search

**Figure 1:**
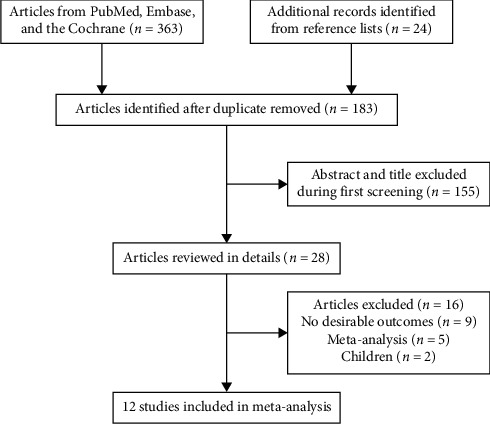
Flow diagram of the literature search and study selection.

A total of 363 studies were identified from the initial electronic database search, and 183 studies remained after removing duplicate publications. Next, 155 studies were further excluded because the research topics were not relevant. The remaining 28 studies were retrieved for full-text evaluation, and 12 RCTs were selected for final analyses [22–33]. Reviewing the reference lists of the retrieved studies yielded 23 potentially included studies, but no new studies met the inclusion criteria (Figure 1).

### 3.2. Study Characteristics

Table 1 summarizes the characteristics of the studies and patients. Overall, a total of 2264 adults with ARDS from 12 RCTs were included in this study, and the sample sizes ranged from 16 to 791. Eight RCTs were conducted in a single country, while four were multicenter studies conducted in two countries. The average age of patients from individual trials ranged from 41.4 to 64.5 years, and the male fraction of patients ranged from 37.5% to 87.5%. Six RCTs included patients that received protective lung ventilation, and the remaining six studies included patients that did not receive protective lung ventilation. Seven of the included trials were of high quality (two studies had Jadad scores of 6, and five studies had Jadad scores of 5), and the remaining five trials were of low quality (three studies had Jadad scores of 4, one study had a score of 3, and the remaining study had a score of 2).

### 3.3. Mortality

**Figure 2:**
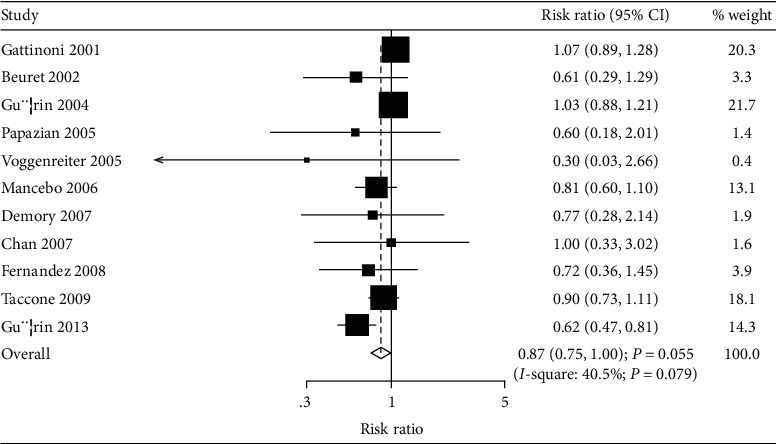
Prone versus supine position ventilation on the risk of mortality.

The effects of prone versus supine position ventilation on the risk of mortality were reported in 11 RCTs. The pooled results suggest that the risk of mortality was reduced by 13% for prone versus supine position ventilation, though this reduced risk was not statistically significant (RR: 0.87; 95% CI: 0.75–1.00; *P* = 0.055; Figure 2). The heterogeneity test indicated potentially significant heterogeneity (*I*^*2*^ = 40.5; *P* = 0.079). Sensitivity analysis indicated prone versus supine positioning might be associated with lower risk of mortality in ARDS patients when excluding the trial conducted by Gattinoni et al. [22] or Guérin et al. [25] (Table 2). Subgroup analyses indicated that prone versus supine positioning was associated with lower risk of mortality if the mean age of patients was <60.0 years, the percentage of male patients was <70.0%, or intervention was used as protective lung ventilation (Table 3). Finally, the interaction *P* test indicated that the treatment effect of prone versus supine positioning on mortality could be affected by the percentage of male patients (*P* = 0.001), and whether used as protective lung ventilation (*P* = 0.012).

### 3.4. Mechanical Ventilation Duration and ICU Stays

**Figure 3:**
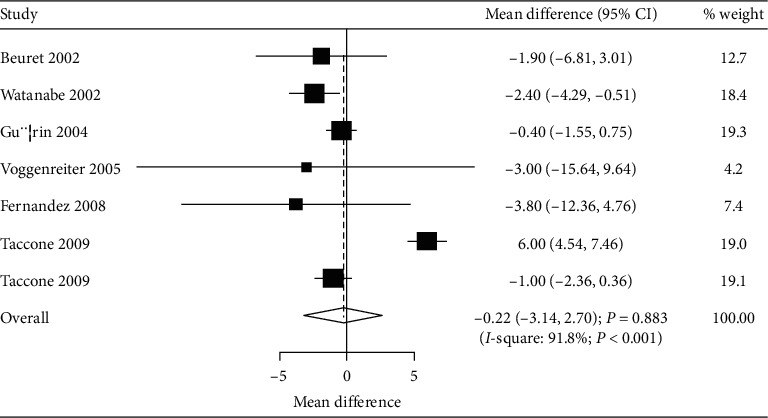
Prone versus supine position ventilation on mechanical ventilation duration.

**Figure 4:**
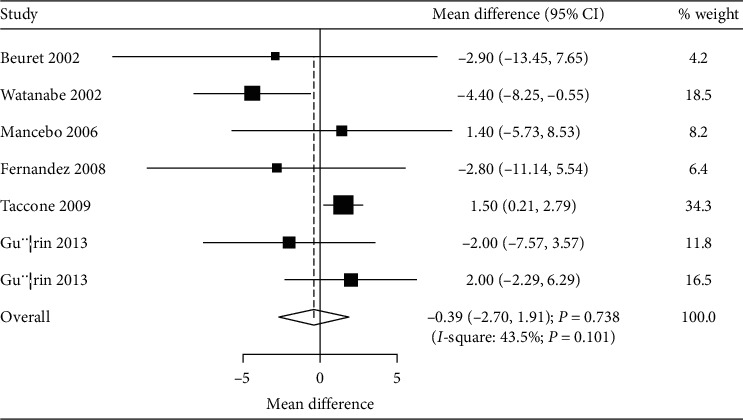
Prone versus supine position ventilation on ICU stays.

**Figure 5:**
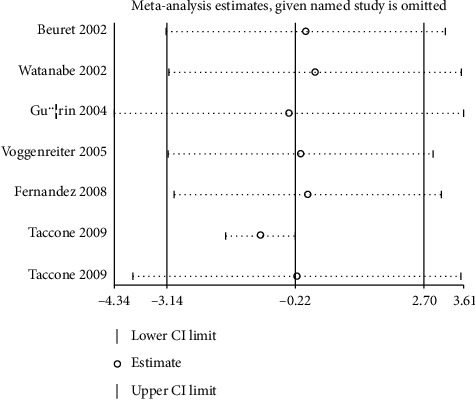
Sensitivity for duration of mechanical ventilation.

**Figure 6:**
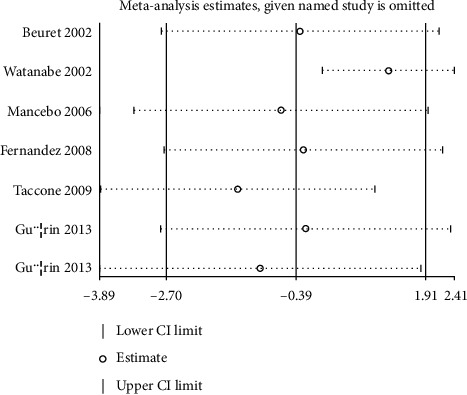
Sensitivity for ICU stays.

The numbers of studies available for mechanical ventilation duration and ICU stays were six (7 cohorts) and six (7 cohorts), respectively. No significant differences between prone and supine positioning on mechanical ventilation duration (WMD: –0.22; 95% CI: –3.14 to 2.70; *P* = 0.883; Figure 3) or ICU stays (WMD: –0.39; 95% CI: –2.70 to 1.91; *P* = 0.738; Figure 4) were detected. There was significant heterogeneity for the duration of mechanical ventilation (*I*^*2*^ = 91.8; *P* < 0.001), while insignificant heterogeneity was detected for ICU stays (*I*^*2*^ = 43.5; *P* = 0.101). The sensitivity analyses indicated that prone versus supine positioning might be associated with shorter mechanical ventilation duration and longer ICU stays (Figures 5 and 6).

### 3.5. Adverse Events

The risks of adverse events between prone and supine positioning are summarized in Table 4. Overall, patients that received prone position ventilation were associated with greater risk of pressure scores (RR: 1.23; 95% CI: 1.07–1.42; *P* = 0.003), displacement of a thoracotomy tube (RR: 3.14; 95% CI: 1.02–9.69; *P* = 0.047), and endotracheal tube obstruction (RR: 2.45; 95% CI: 1.42–4.24; *P* = 0.001) than those received supine position ventilation. No significant differences between prone and supine positioning were observed for the risks of displacement of tracheal tube (RR: 1.35; 95% CI: 0.47–3.84; *P* = 0.579), unplanned extubation (RR: 1.02; 95% CI: 0.73–1.43; *P* = 0.906), selective intubation (RR: 2.64; 95% CI: 0.26–26.73; *P* = 0.411), loss of venous access (RR: 1.52; 95% CI: 0.22–10.26; *P* = 0.669), hemoptysis (RR: 0.85; 95% CI: 0.35–2.05; *P* = 0.717), cardiac arrest (RR: 0.71; 95% CI: 0.40–1.26; *P* = 0.245), pneumothorax (RR: 0.86; 95% CI: 0.58–1.29; *P* = 0.471), and ventilator-associated pneumonia (RR: 1.34; 95% CI: 0.65–2.76; *P* = 0.427).

### 3.6. Publication Bias

**Figure 7:**
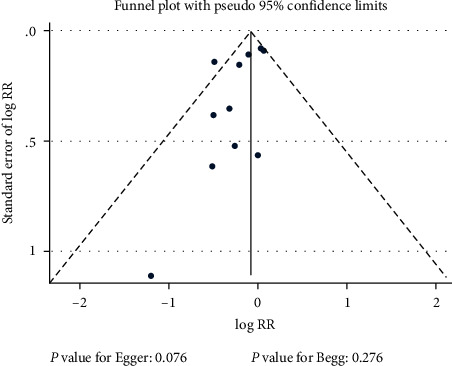
Funnel plot for mortality.

**Figure 8:**
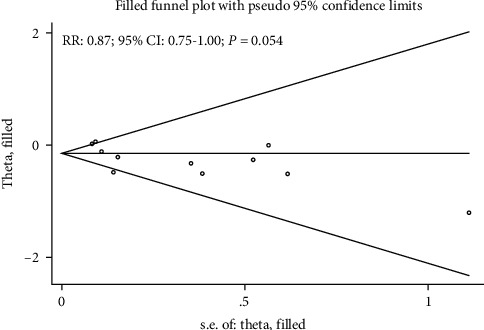
Trim and fill method for mortality.

Publication bias for mortality was assessed by funnel plots, Egger's test, and Begg's test, and the results suggest potential publication bias for mortality (*P* value for Egger's test: 0.076; *P* value for Begg's test: 0.276; Figure 7). The conclusions were not changed after adjustment for publication bias by using the trim and fill method (RR: 0.87; 95% CI: 0.75–1.00; *P* = 0.054; Figure 8) [34].

## 4. Discussion

Mechanical ventilation is widely used to improve oxygenation and reduce harmful effects in ARDS patients, though whether prone positioning during ventilation can improve clinical endpoints versus supine positioning remains unclear. Several previous studies have suggested that future RCTs should be conducted with bigger sample sizes, and the current meta-analysis represents the best current evidence regarding the efficacy and safety of prone versus supine positioning in mechanical ventilation of patients with ARDS. These quantitative analyses contained 2264 adults with ARDS across a broad range of patient characteristics. Our findings indicate that ARDS patients that underwent ventilation with prone positioning might experience lower risk of mortality, shorter mechanical ventilation duration, and longer ICU stays, although the pooled effect estimates suggest no significant differences between groups. Moreover, the risk of pressure scores, displacement of a thoracotomy tube, and endotracheal tube obstruction were significantly increased in ARDS patients received prone positioning. Finally, the treatment effectiveness of prone versus supine positioning on the risk of mortality could affect by percentage male, and whether used as protective lung ventilation.

A meta-analysis conducted by Alsaghir and Martin contained five studies and found that prone positioning did not yield additional benefits with regard to mortality, whereas it improved oxygenation as compared with supine positioning. Moreover, prone positioning might be associated with lower risk of mortality for patients with higher illness severity [35]. A meta-analysis conducted by Sud et al. involved 9 RCTs and found prone ventilation was associated with a reduced risk of mortality in patients with severe hypoxemia [6]. In 2014, they update this study and contained 11 RCTs. They point out prone positioning could improve mortality for ARDS patients that received protective lung ventilation [36]. A study by Hu et al. included 9 RCTs and suggested that prone versus supine positioning was associated with lower risk of mortality in patients with severe ARDS, high PEEP levels, or who received long-term prone positioning [37]. That meta-analysis of 11 RCTs indicated that prone position ventilation significantly reduced the risk of mortality in severe ARDS patients or in patients who received sufficient duration of prone positioning. Moreover, patients that received prone positioning could had increased risk of pressure ulcers and major airway problems [38]. Mora-Arteaga et al. identified 7 RCTs and found that prone position ventilation could decrease mortality risk for patients with low tidal volume, prolonged pronation, starting within the first 48 hours of disease evolution, and severe hypoxemia [39]. Munshi et al. conducted a meta-analysis of 8 RCTs and found that prone positioning is associated with lower risk of mortality among patients with moderate to severe ARDS, or applied prone positioning for at least 12 hours daily [40]. However, the limitations of these studies included several other efficacy and safety outcomes were not calculated, or subgroup analyses for the risk of mortality according to other patients' characteristics were not presented. Therefore, the present systematic review and meta-analysis was conducted to evaluate the efficacy and safety of prone versus supine positioning for ARDS patients.

The summary results indicate that prone versus supine positioning was not associated with risk of mortality, though this conclusion was not stable and could have been affected by two specific individual trials [22, 25]. Moreover, we noted that prone versus supine positioning was associated with lower risk of mortality when the mean age of the patients was <60.0 years, the percentage of male patients was <70.0%, or intervention was used as protective lung ventilation. The potential reasons for this included (1) prone positioning could decrease the risk of lung injury causes by stress and strain forces [6, 41]; (2) severe ARDS is associated with excess risk of lung injury from shear and strain force due to a low ratio of well-aerated lung tissues to poorly aerated or nonaerated lung tissues [42]; (3) treatment effectiveness is greater in younger ARDS patients than in elderly ARDS patients which could be explained by the difference of the disease severity, which could affect the prognosis for patients with ARDS; (4) the result of subgroup analyses indicates that the beneficial effects on mortality in females might be explained by lifestyle factors and the severity of disease, whereas this result is based on male proportion, and this analysis just provides a relative result; and (5) the use of protective lung ventilation was associated with lower lung injury risk through minimizing tidal volumes and optimizing PEEP [43, 44].

The pooled results of this study indicate no significant differences between prone and supine positioning for mechanical ventilation duration and ICU stays. These conclusions are not stable and could be altered by excluding individual trials. The study conducted by Taccone et al. indicated that survival of patients that received prone positioning was significantly longer mechanical ventilation duration than supine positioning, which could be due to the fact that protocols of mechanical ventilation differed across the included studies [32]. Moreover, the duration of mechanical ventilation and ICU stays were significantly correlated with the severity of ARDS, which could affect the prognosis of patients with ARDS. Furthermore, the heterogeneity across included trials for mechanical ventilation duration, which could be explained by various characteristics and disease status for included patients. The adverse events are also summarized between prone and supine positioning for ARDS patients. We noted that prone positioning was associated with greater risk of pressure scores, displacement of a thoracotomy tube, and endotracheal tube obstruction. However, these results were based on a smaller number of included trials, and this result needs to be verified by a large-scale RCT.

Several strengths of this study should be highlighted: (1) the selection and concerning confounder biases were lower because this analysis was based on RCTs; (2) this study utilized a large sample size, and the results are more robust than individual trials; and (3) stratified analyses based on patients' characteristics were conducted, which allows us to obtain more exploratory results. However, several limitations should also be acknowledged: (1) substantial heterogeneity was detected for several outcomes, which could not be interpreted in subgroup analyses; (2) the analysis of this study was based on published articles, and unpublished data were not available; and (3) the background therapies for ARDS patients were not known, which also affect the prognosis of ARDS.

## 5. Conclusion

The findings of this study indicate that prone positioning might play an important role on the risk of mortality, especially for patients <60.0 years old, percentage male <70.0%, or intervention used with protective lung ventilation. Moreover, there were no significant differences between prone and supine positioning for mechanical ventilation duration and ICU stays. ARDS patients that received prone position ventilation could experience increased risk of pressure scores, displacement of a thoracotomy tube, and endotracheal tube obstruction. These findings should be verified by further large-scale RCTs.

## Figures and Tables

**Table 1:** Baseline characteristics of the included studies.

Study	Country	Sample size	Mean age (years)	Percentage male (%)	Mean PaO_2_: FIO_2_	Mean PEEP (cm H_2_O)	Mean FIO_2_ (%)	Duration of ARDS	Duration of prone positioning	Protective lung ventilation	Jadad scale
Gattinoni et al. [22]	Italy and Switzerland	304	58.0	70.4	125/130	9.0	73.0	NA	7 hours daily for 4.7 days	No	6
Beuret et al. [23]	France	51	55.0	70.6	315/337	NA	NA	<24.0 hours	4 hours daily for 6.0 days	No	5
Watanabe et al. [24]	Japan	16	64.5	87.5	166/NA	NA	NA	NA	6 hours daily for 4.0 days	No	3
Guérin et al. [25]	France	791	62.2	75.0	150/155	8.0	66.0	12.0–24.0 hours	9 hours daily for 4.1 days	No	5
Papazian et al. [26]	France	26	53.0	65.4	101/106	11.5	71.5	<24.0 hours	12 hours daily for 1.0 day	No	2
Voggenreiter et al. [27]	Germany	40	41.4	82.5	215/228	11.5	49.0	<48.0 hours	11 hours daily for 7.0 day	Yes	5
Mancebo et al. [28]	Spain and Mexico	136	54.0	63.2	132/161	7.0	82.0	<48.0 hours	17 hours daily for 10.1 day	No	4
Demory et al. [29]	France	30	54.0	73.3	122.0^*∗*^	11.0	NA	<24.0 hours	12 hours daily for 1.0 day	Yes	4
Chan et al. [30]	Taiwan	22	62.3	81.8	111/108	13.3	87.5	<72.0 hours	24 hours daily for 4.4 day	Yes	4
Fernandez et al. [31]	Spain	40	54.6	37.5	114/122	11.2	84.5	<48.0 hours	18 hours daily for first 2 days	Yes	5
Taccone et al. [32]	Italy and Spain	342	60.0	71.3	113.0^*∗*^	10.0	72.0	<72.0 hours	18 hours daily for first 8.3 days	Yes	5
Guérin et al. [33]	France and Spain	466	59.0	68.2	100/100	10.0	79.0	<36.0 hours	17 hours daily for first 4.0 days	Yes	6

^*∗*^Both group; ARDS: acute respiratory distress syndrome; FIO_2_: fractional concentration of inspired oxygen; NA: not available; PaO_2_: partial pressure of arterial oxygen; PEEP: positive end-expiratory pressure.

**Table 2:** Sensitivity analysis for mortality.

Excluding study		RR and 95% CI	*P* value	Heterogeneity (%)	*P* value for heterogeneity
Gattinoni 2001		0.83 (0.71–0.97)	0.017	31.2	0.159
Beuret 2002		0.88 (0.76–1.02)	0.084	42.2	0.076
Guérin 2004		0.82 (0.69–0.98)	0.026	37.3	0.111
Papazian 2005		0.87 (0.75–1.01)	0.068	44.8	0.061
Voggenreiter 2005		0.87 (0.75–1.01)	0.064	42.8	0.073
Mancebo 2006		0.87 (0.74–1.03)	0.099	44.2	0.064
Demory 2007		0.87 (0.74–1.01)	0.064	46.0	0.054
Chan 2007		0.86 (0.74–1.00)	0.055	46.4	0.052
Fernandez 2008		0.87 (0.75–1.01)	0.076	44.9	0.060
Taccone 2009		0.85 (0.71–1.01)	0.072	46.5	0.052
Guérin 2013		0.96 (0.88–1.06)	0.458	0.0	0.579

**Table 3:** Subgroup analysis for mortality.

Factor	Subgroups	Number of studies	RR and 95% CI	*P* value	Heterogeneity (%)	*P* value for heterogeneity	*P* value between subgroups
Sample size	≥100	5	0.89 (0.74–1.06)	0.198	70.0	0.010	0.135
	<100	6	0.69 (0.47–1.02)	0.064	0.0	0.942	

Mean age (years)	≥60.0	3	0.98 (0.86–1.12)	0.768	0.0	0.608	0.245
	<60.0	8	0.77 (0.60–0.99)	0.042	51.6	0.044	

Percentage male (%)	≥70.0	7	0.99 (0.90–1.10)	0.906	0.0	0.582	0.001
	<70.0	4	0.70 (0.58–0.85)	<0.001	0.0	0.595	

Duration of intervention	≥3.0 days	9	0.87 (0.74–1.02)	0.079	50.5	0.040	0.466
	<3.0 days	2	0.69 (0.32–1.51)	0.358	0.0	0.758	

Protective lung ventilation	Yes	6	0.77 (0.63–0.93)	0.006	10.7	0.347	0.012
	No	5	0.98 (0.86–1.12)	0.759	18.0	0.300	

Study quality	High	7	0.87 (0.72–1.05)	0.139	61.2	0.017	0.329
	Low	4	0.81 (0.61–1.06)	0.119	0.0	0.943	

**Table 4:** The summary results for adverse events.

Outcomes	RR and 95% CI	*P* value	Heterogeneity (%)	*P* value for heterogeneity
Pressure sores	1.23 (1.07–1.42)	0.003	0.0	0.589
Displacement of tracheal tube	1.35 (0.47–3.84)	0.579	73.3	0.053
Displacement of a thoracotomy tube	3.14 (1.02–9.69)	0.047	0.0	0.470
Unplanned extubation	1.02 (0.73–1.43)	0.906	8.6	0.350
Selective intubation	2.64 (0.26–26.73)	0.411	58.9	0.119
Endotracheal tube obstruction	2.45 (1.42–4.24)	0.001	0.0	0.750
Loss of venous access	1.52 (0.22–10.26)	0.669	90.8	0.001
Hemoptysis	0.85 (0.35–2.05)	0.717	66.0	0.086
Cardiac arrest	0.71 (0.40–1.26)	0.245	70.9	0.064
Pneumothorax	0.86 (0.58–1.29)	0.471	0.0	0.677
Ventilator-associated pneumonia	1.34 (0.65–2.76)	0.427	0.0	0.499

## Data Availability

The data used to support the findings of this study are included within the article.
